# Metabolic adaptation fluctuates with different prediction equations: a secondary analysis based on a weight-loss clinical trial

**DOI:** 10.3389/fnut.2025.1543263

**Published:** 2025-08-29

**Authors:** Molian Tang, Jialu Wang, Yi Xiang, Renying Xu

**Affiliations:** ^1^Department of Clinical Nutrition, Ren Ji Hospital, Shanghai Jiao Tong University School of Medicine, Shanghai, China; ^2^Department of Nutrition, College of Health Science and Technology, Shanghai Jiao Tong University School of Medicine, Shanghai, China; ^3^Department of Clinical Nutrition, Xin Hua Hospital Affiliated to Shanghai Jiao Tong University School of Medicine, Shanghai, China

**Keywords:** metabolic adaption (MA), resting metabolic rate (RMR), Katch-McArdle, bioelectric impedance analysis (BIA), weight management

## Abstract

**Objective:**

Metabolic adaption (MA) might be of clinical relevance in weight loss management. However, it is unclear whether resting metabolic rate (RMR) reduction in weight loss reflects true MA or fat free mass (FFM) loss.

**Methods:**

We re-analyzed the data based on a weight loss trial of 131 patients (aged 33.3 ± 6.7 years) with overweight/obesity. Anthropometric data, body composition, daily physical activity, sleep hour and dietary intake were collected for every 4 weeks (baseline, week 4, 8, 12, and 16). The linear mixed model was used to evaluate the absolute change in RMR and adjusted RMR [aRMR = RMR (kcal) divided by FFM (kg)] with two different equations (Katch-McArdle-determined vs. BIA-determined) for every 4 weeks after adjustment of age, sex, daily physical activity, sleep hours, dietary intake, and baseline FFM and fat mass (FM).

**Results:**

Following the 16-week intervention, a significant reduction was observed in body weight [β: −5.6 kg; 95% Confidence Interval (CI): −6.3 kg, −5.0 kg], BMI (β: −2.3 kg/m^2^; 95%CI: −2.5 kg/m^2^, −2.0 kg/m^2^), FM (β: −4.7 kg; 95%CI: −5.2 kg, −4.1 kg), and FFM (β: −0.9 kg; 95%CI: −1.2 kg, −0.7 kg). Both Katch-McArdle-determined RMR and BIA-determined RMR presented a significant decrease between baseline and the end of the intervention (week 16). A small but statistically significant increase in Katch-McArdle-determined aRMR (β: 0.19 kcal/kg; 95%CI: 0.14 kcal/kg, 0.23 kcal/kg; adjusted *p*-value <0.0001) was confirmed by linear mixed models. While BIA-determined aRMR generally showed decreasing trends across the follow-up periods, only Week 12 demonstrated a statistically significant inverse association compared with the baseline (β: −0.21 kcal/kg; 95% CI: −0.29 kcal/kg, −0.13 kcal/kg, adjusted *p*-value = 0.01).

**Conclusion:**

The use of different prediction equations might account for variations in MA in this study. The results highlight the importance of preserving FFM during weight loss, thus to prevent reductions in RMR.

## Introduction

Metabolic adaptation (MA) is a contentious concept in which the body reduces resting metabolic rate (RMR) to facilitate weight regain after intentional weight loss (1). Since RMR accounts for ~70% of total daily energy expenditure, it is hypothesized that establishing the importance of MA would facilitate strategies to prevent body weight regain (2).

To date, the nature of MA remains unclear, with some studies underscore the importance (3–7) while others not (8, 9), establishing its existence facilitates different strategies and nutritional interventions, thus preventing body weight regain.

Moreover, it is important to distinguish whether the reduction in RMR was caused by MA or by the decrease in fat free mass (FFM). Müller et al. demonstrated that the decline in FFM (~65%) and MA (~35%) in combine could contribute to a reduction in RMR during the early phase of calorie restriction (10). FFM, consisting of metabolically active tissues and low-metabolic-rate tissues or organs (11), is primary determinant of RMR (12), a large reduction in FFM is contributed to slow the metabolic rate. However, intentional weight loss in people with obesity primarily decreases body fat, but also decreases FFM (13). Increasing protein intake from 0.8 g/kg/day to 1.2 g/kg/day reduced the weight loss-induced decline in FFM by ~45% (14). Hence, it is important to maintain the FFM with a higher daily protein intake (15). Another critical issue is the variability in RMR prediction equations across the studies (3, 6, 7), which may account for the observed subtle RMR changes (~50 kcal/day after a 12 kg weight loss) (16). Therefore, overestimation of RMR may lead to excessive caloric restriction, potentially compromising treatment adherence and lean mass preservation. On the other hand, underestimation of RMR may increase the likelihood of early treatment termination. Given accurate RMR estimation is clinically critical, verifying the accuracy of MA represents a fundamental research priority.

Herein, we conducted a secondary analysis based on our previous 16-week randomized clinical trial in Chinese overweight/obesity adults (17). The purpose of this study is to determine the differences in RMR and FFM adjusted RMR (aRMR = RMR/FFM) (15) during weight loss by comparing two prediction RMR equation (Katch-McArdle and BIA-derived equations) used in the study.

## Methods

### Study protocol

This was a secondary analysis based on an open-label, randomized, parallel clinical trial performed in two teaching hospitals (Clinical Registration Number: ChiCTR2100042637). All the participants were Chinese adults (18–65 years old) with overweight and obesity [Body mass index (BMI) between 24.0 kg/m^2^ to 35.0 kg/m^2^ (18) and waist circumference (WC) ≥90 cm for males and 80 cm for females]. The inclusive and exclusive criteria was described in the previous study (17) and in the Supplementary material. Briefly, a screening (week −1), a baseline (week 0), and four follow-up visits (week 4, 8, 12, and 16) were performed for all the participants (19). The study was performed by both the Ethics Committee, Ren Ji Hospital (KY2020-204) and Xin Hua Hospital (XHEC-C-2020-014-3), Shanghai Jiao Tong University School of Medicine. Written consents were obtained from each participant.

### Data collection

Anthropometric data and body composition were evaluated at baseline (Week 0) and every four weeks (week 4, 8, 12, and 16) throughout the study. Body weight and composition [fat mass (FM) and FFM] were evaluated using bioelectric impedance analysis (BIA) (20) (Tanita Corporation, MC-180, Tokyo, Japan). To test whether changes in RMR were caused by the prediction model, we used Katch-McArdle equation (21) to calculate RMR and compared it with BIA-determined RMR.

The target of daily energy intake was generated by RMR (based on the Katch-McArdle equation) multiple the factor of physical activity (=1.25), then minus 500 kcal, and the daily protein recommendation was ≈2.0 g/kg FFM. The Katch-McArdle prediction equation was shown as follows: RMR = [370 + 21.6^*^lean body mass (kg)]. All the participants received two servings of a commercial meal replacement product (Herbalife China Protein Drink Mix dry powder, the nutrient composition in Supplementary Table 1), with one serving at breakfast and one at either lunch or dinner. An online (WeChat app) 3-day dietary recall was used to record daily food intake at each follow-up visit. Daily physical activity (quantified as step counts) and sleep hours were monitored by an electronic wearable device (the Mi Smart Band 4, Xiaomi Technology Co. LTD, Beijing, China). A total number of 10,000 steps per day was recommended for all the participants.

### Outcome assessment

The primary outcome was the absolute change in RMR and adjusted RMR [aRMR = RMR (kcal) divided by FFM (kg)] from baseline (Week 0) to the end of the trial (Week 16). The residuals (rRMR) between the measured RMR (BIA-determined) and the prediction RMR (Katch-McArdle equation) were calculated as well (22). The secondary outcomes were absolute changes in body weight, FM, FFM, waist and hip circumferences before and after the trial.

### Statistical analyses

Initial power calculation was conducted using parameters from previous published studies (23, 24). A total number of 16–28 participants is determined enough to provide 80% power to detect statistically significant change in body weight at a two-tailed significance level of 0.05. However, 100 participants were proposed for the study because the Chinese regulation required at least 100 participants for a weight loss clinical trial to substantiate the effectiveness. Assuming that the attrition rate was 35% after the intervention, the final estimated sample size was 154 while 100 of them remained at the end of the intervention (17).

Continuous variables were reported as mean and standard deviation (SD), after testing for normality with the Kolmogorov-Smirnov test. Categorical variables were presented as counts and percentages.

We used linear mixed models with time as a fixed effect and patients as a random effect to evaluate absolute changes in both RMR and adjusted RMR (aRMR = RMR [kcal]/FFM [kg]) (15) derived from two prediction equations (Katch-McArdle equation vs. BIA-based equation) at 4-week intervals. Models were adjusted for age, sex, daily physical activity, sleep hours, dietary intake, and baseline FFM and fat mass (FM) (2). A Tukey-Kramer correction was applied for *post hoc* comparisons.

Interaction effects between sex and week were tested by incorporating cross-product terms in the multivariable model. The association between changes in aRMR and FM was assessed using generalized linear modeling. Assumption of linearity of RMR and FFM at 4-week intervals were confirmed (Supplementary Figure 1). To verify the robustness of our primary findings, we performed three sensitivity analyses: exclusion of outliers identified by Tukey's fences (± 2.7 interquartile range); sex-stratified analysis (males vs. females) and age-stratified analysis (18–35 years vs. 36–65 years). All statistical analyses were conducted using Statistical Analysis System (SAS) 9.4 software (SAS Institute, Inc, Cary, NC). A two-tailed *p* < 0.05 was considered as statistical significance.

## Results

### Basic characteristics

A total number of 131 (38 males and 93 females) participants was included. The average age was 33.3 ± 6.7 years while the average BMI, WC, and hip circumference were 28.4 kg/m^2^, 97.3 cm and 101.9 cm. The age, baseline BMI, and WC were not significant between males and females while females had a higher proportion of FM and a lower proportion of FFM than males (Table 1).

**Table 1:** Baseline characteristics of 131 participants with overweight and obesity.

**Parameters**	**Male (*n* = 38)**	**Female (*n* = 93)**	***p*-value**
Age, years	32.9 ± 7.0	33.4 ± 6.6	0.65
Body weight, kg	87.0 ± 10.1	74.9 ± 8.9	< 0.001
Height, cm	174.4 ± 5.4	162.4 ± 5.5	< 0.001
BMI, kg/m^2^	28.5 ± 2.4	28.4 ± 3.0	0.84
Body fat, kg	24.4 ± 6.2	29.5 ± 6.2	< 0.001
Fat free mass, kg	62.6 ± 6.1	45.4 ± 4.1	< 0.001
Waist circumference, cm	95.8 ± 5.9	97.9 ± 5.9	0.09
Hip circumference, cm	99.9 ± 5.0	102.8 ± 5.4	0.0066
Katch-McArdle determined RMR, kcal	1723.2 ± 132.8	1350.5 ± 88.9	< 0.001
Katch-McArdle determined aRMR, kcal/kg	27.6 ± 0.6	29.8 ± 0.7	< 0.001
BIA determined RMR, kcal	1,756.4 ± 165.6	1,380.3 ± 104.0	0.0003
BMI determined aRMR, kcal/kg	28.1 ± 0.8	30.5 ± 1.2	< 0.0001
Systolic BP, mmHg	122.1 ± 12.7	111.2 ± 12.3	< 0.001
Diastolic BP, mmHg	76.6 ± 10.5	69.5 ± 9.2	0.0002

BMI, body mass index; BP, blood pressure; RMR, resting metabolic rate; aRMR, adjusted resting metabolic rate.

RMR and body composition were determined by bioimpedance analysis. Adjusted RMR was calculated by RMR (kcal) divided by fat-free mass (kg).

Katch-McArdle prediction equation: RMR = 370 + 21.6^*^lean body mass (kg).

BIA determined RMR based on Tanita MC-180 (Tanita Corporation, MC-180, Tokyo, Japan).

### Change in body composition

There was a significant decrease in body weight [β: −5.6 kg; 95% Confidence Interval (CI): −6.3 kg, −5.0 kg], BMI (β: −2.3 kg/m^2^; 95%CI: −2.5 kg/m^2^, −2.0 kg/m^2^), FM (β: −4.7 kg; 95%CI: −5.2 kg, −4.1 kg), and FFM (β: −0.9 kg; 95%CI: −1.2 kg, −0.7 kg).

### Change in RMR and aRMR

A significant decrease in Katch-McArdle-determined RMR (β: −20.4; 95%CI: −26.4 kcal, −14.5 kcal; adjusted *p*-value < 0.001) while a small but statistically significant increase in aRMR (β: 0.19 kcal/kg; 95%CI: 0.14 kcal/kg, 0.23 kcal/kg; adjusted *p*-value < 0.0001) was confirmed by linear mixed models (Table 2). While BIA-determined aRMR generally showed decreasing trends across follow-up periods, only Week 12 demonstrated a statistically significant inverse association (β: −0.21; 95% CI: −0.29, −0.13, adjusted *p*-value = 0.01; Table 2).

**Table 2:** Differences in body weight, body mass index, body composition, and resting metabolic rate before and after the study.

**Parameters**		**Follow up**				
		**Baseline**	**Week 4**	**Week 8**	**Week 12**	**Week 16**
BW	β (95%CI)	0	−2.3 (−2.9, −1.7)	−3.8 (−4.4, −3.2)	−4.9 (−5.5, −4.3)	−5.6 (−6.3, −5.0)
	Adjusted *p*-value	N/A	< 0.0001	< 0.0001	< 0.0001	< 0.0001
BMI	β (95%CI)	0	−0.9 (−1.1, −0.6)	−1.4 (−1.7, −1.2)	−1.9 (−2.1, −1.6)	−2.3 (−2.5, −2.0)
	Adjusted *p*-value	N/A	< 0.0001	< 0.0001	< 0.0001	< 0.0001
FM	β (95%CI)	0	−1.9 (−2.4, −1.4)	−3.1 (−3.6, −2.6)	−4.0 (−4.6, −3.5)	−4.7 (−5.2, −4.1)
	Adjusted *p*-value	N/A	< 0.0001	< 0.0001	< 0.0001	< 0.0001
FFM	β (95%CI)	0	−0.4 (−0.6, −0.1)	−0.7 (−0.9, −0.4)	−0.9 (−1.1, −0.6)	−0.9 (−1.2, −0.7)
	Adjusted *p*-value	N/A	0.0002	< 0.0001	< 0.0001	< 0.0001
Katch-McArdle^a^ determined RMR, kcal	β (95%CI)	0	−7.6 (−12.9, −2.4)	−14.3 (−19.8, −8.8)	−19.0 (−24.7, −13.3)	−20.4 (−26.4, −14.5)
	Adjusted *p*-value	N/A	0.0002	< 0.0001	< 0.0001	< 0.0001
Katch-McArdle determined aRMR, kcal/kg	β (95%CI)	0	0.07 (0.03, 0.11)	0.13 (0.09, 0.18)	0.17 (0.13, 0.21)	0.19 (0.14, 0.23)
	Adjusted *p*-value	N/A	0.002	< 0.0001	< 0.0001	< 0.0001
BIA-determined^b^ RMR, kcal	β (95%CI)	0	−13.8 (−20.8, −6.9)	−25.2, (−32.5, −18.0)	−34.2 (−41.7, −26.6)	−36.1 (−43.9, −28.3)
	Adjusted *p*-value	N/A	0.0001	< 0.0001	< 0.0001	< 0.0001
BIA-determined aRMR, kcal/kg	β (95%CI)	0	−0.09 (−0.16, −0.01)	−0.14 (−0.22, −0.06)	−0.21 (−0.29, −0.13)	−0.20 (−0.28, −0.10)
	Adjusted *p*-value	N/A	0.88	0.37	0.01	0.15
rRMR, kcal	β (95%CI)	N/A	−6.8 (−10.1, −3.5)	−11.5 (−15.0, −8.1)	−16.1 (−19.7, −12.5)	−16.1 (−19.8, −12.4)
	Adjusted *p*-value		0.008	< 0.0001	< 0.0001	< 0.0001
a_rRMR, kcal/kg	β (95%CI)		−0.16 (−0.23, −0.08)	−0.26 (−0.34, −0.19)	−0.37 (−0.45, −0.29)	−0.37 (−0.46, −0.29)
	Adjusted *p*-value		0.03	< 0.0001	< 0.0001	< 0.0001

Data was presented as β (95% confidence interval).

BW, body weight; BMI, body mass index; FM, fat mass; FFM, free fat mass, RMR, resting metabolic rate; aRMR, adjusted RMR; rRMR, the residual RMR; a_rRMR, adjusted rRMR.

RMR and body composition were determined by bioimpedance analysis. Adjusted RMR was calculated by RMR (kcal) divided by fat-free mass (kg). The rRMR was calculated between the measured RMR (BIA-determined) and the prediction RMR (Katch-McArdle equation). Adjusted rRMR was calculated by rRMR (kcal) divided by fat-free mass (kg).

^a^Katch-McArdle prediction equation: RMR = 370 + 21.6^*^lean body mass (kg).

^b^BIA-determined RMR based on Tanita MC-180 (Tanita Corporation, MC-180, Tokyo, Japan).

Changes over time were analyzed using linear mixed-effects models with time as a fixed effect, participant as a random effect, and adjusted for age, sex, physical activity, sleep hour, dietary intake, and baseline FFM and FM, followed by *post hoc* comparisons between time points with Tukey-Kramer adjustment. All these parameters obtained at each follow-up visit were considered as repeated measurements.

Residual RMR (rRMR) showed progressive and significant decrease from baseline across all follow-ups (all adjusted *p*-value < 0.05), with similar significant reduction observed for FFM-adjusted rRMR (adjusted *p*-value < 0.05; Table 2).

### Sensitivity analysis

Sensitivity analysis consistently demonstrated significant reduction in both absolute and FFM-adjusted RMR across follow-ups (adjusted *p*-value < 0.05 for Katch-McArdle determined), except for BIA-determined FFM-adjusted RMR (Supplementary Table 2).

### The association between the difference in body composition and aRMR

The difference in FFM was significantly associated with the difference in aRMR (by Katch-McArdle equation) at week 4, 8, 12, and 16. In contrast, changes in fat mass (FM) were negligible in the early stages but reached statistical significance by Week 16, though the effect size remained small, indicating the greater decrease in FFM, the greater reduction in aRMR (Supplementary Table 3).

## Discussions

In the current study, we observed significant decreases in body weight, BMI, FM, FFM, and RMR after 16-week intervention. However, RMR per kilogram of FFM (Katch-McArdle determined) increased while both residual RMR and FFM-adjusted rRMR (a_rRMR) decreased.

FFM [consisting of metabolically active FFM and low-metabolic-rate tissues or organs (11)] is a primary determinant of RMR. A substantial loss of FFM leads to a decreased metabolic rate. However, reductions in FFM are commonly observed in weight-loss studies, contributing to 25%−40% of total body weight reduction (13, 25). Our findings demonstrated a significant increase in RMR per kilogram of FFM (β = 0.19 kcal/kg, 95%CI:0.14 kcal/kg, 0.23 kcal/kg) after 16-week intervention, indicating greater metabolic efficiency of FFM components—most notably skeletal muscle [account for 50% of FFM (13)] (12). However, the residual RMR and a_rRMR significantly decreased after intervention, indicating substantial MA was present. The underlying mechanism why MA happens during weight loss remains unclear. First of all, MA might be caused by different statistical methods, in other words, discrepancies among different regression prediction equations (26). Further, thyroid suppression, disproportionate loss of high metabolic-rate organs, and altered adiponectin signaling during weight loss are possible reasons for MA (12).

The key point in managing obesity and its related chronic diseases is loss of body fat, but not FFM, especially skeletal muscle. The loss of FFM is obviously associated with a series of negative symptoms, including fatigue, osteoporosis, and high risk of fall as well as worsen insulin resistance (27). Maintaining or increasing skeletal muscle is crucial because it is the main organ responsible for uptake of serum glucose after meal. On the other hand, more muscle mass is helpful to maintain insulin sensitivity by alleviating the workload on pancreatic β-cells and to lower free fatty acid levels (28). Optimizing body composition through muscle quality not only helps preserve active physical function but also promotes better metabolic regulation, leading to long-term health benefits (29).

### Strength and limitation

The main strength lies in controlling for important confounding variables affecting weight loss, including dietary intake, daily physical activities, as well as age, sex and baseline FFM/FM (2). The primary limitation of this study is that RMR was estimated using two prediction equations rather than measured via indirect calorimetry. Further, BIA has some known limitations, including applicability only to specific populations, vulnerability to hydration fluctuations, and less precise in skeletal muscle quantification than gold-standard methods (30). These factors could explain, in part, the divergent outcomes between prediction equations in our analysis. Secondly, the inclusion and exclusion criterion for the current study were adapted from an established research protocol (as stated in the methodology section), therefore, the generalizability of our conclusions is limited. Future studies need to be performed in different populations to reduplicate our results. Further, the intensity and type of exercise were unavailable, which was closely associated with the strength of muscle mass.

## Conclusions

The results of the current study showed that different prediction equations might lead to discrepancies in predicted RMR after adjustment of FFM. A relatively high protein diet combined with moderate exercise could be helpful to maintain both muscle mass and mass strength during weight loss.

## Data Availability

The raw data supporting the conclusions of this article will be made available by the authors, without undue reservation.
