# Supplementary material for: Metabolic adaptation fluctuates with different prediction equations: a secondary analysis based on a weight-loss clinical trial
Source: Front Nutr. 2025 Aug 29;12:1543263. doi: 10.3389/fnut.2025.1543263 (PMC12425712; doi:10.3389/fnut.2025.1543263)
Supplement: Supplementary file 1 [file Data_Sheet_1.docx]

**The criteria for inclusion and exclusion**

◾Inclusion criteria:

(1). No history of hypertension, diabetes, and gout;

(2). systolic blood pressure < 160 mmHg or diastolic blood pressure < 100 mmHg;

(3). fasting blood glucose < 7.0 mmol/L or glycated hemoglobin A1c < 6.5%;

(4). triglyceride ≤2.2 mmol/L;

(5). fasting serum level of uric acid < 728.0 μmol/L (1 mg/dl≈59.5 μmol/L) for males and 625.0 μmol/L for females;

(6). Serum level of aspartate transferase < 35 U/L and alanine transferase < 36 U/L;

(7). Fasting serum level of low-density lipoprotein cholesterol <3.36 mmol/L (1mmol/L=1mg/dL×0.02586).

◾Exclusion criteria:

(1). Those who participated in another clinical trial within the past 12 months;

(2). Those enrolled in other clinical intervention programs;

(3). Those receiving any other anti-obesity interventions;

(4). Pregnancy women or lactation;

(5). Those with medical conditions which might prevent him/her from fulfilling the study requirements.

**Supplementary Table 1**. Food labels for Herbalife China Protein Drink Mix dry powder

|  | Label Claim | NRV |
| --- | --- | --- |
| Energy | 387KJ | 5% |
| Protein | 10g | 17% |
| Carb (excl. fiber) | 9g | 3% |
| Fiber | 2g | 8% |
| Fat | 1.3g | 2% |
| Na | 180mg | 9% |
| Vitamin D | 0.3ug | 6% |
| Vitamin E (α-TE) | 2.5mg | 18% |
| Vitamin B_1_ | 0.32-0.4mg* | 23-29% |
| Vitamin B_2_ | 0.32-0.4mg* | 23-29% |
| Vitamin B_6_ | 0.3-0.4mg* | 21-29% |
| Vitamin B_12_ | 0.75-1.5ug* | 31-62% |
| Vitamin C | 32.5-50mg* | 32.5-50% |
| Nicotinamide | 4.5-6mg* | 32-43% |
| Folate (DFE) | 62-187ug* | 16-47% |
| Pantothenic acid | 1.05-1.8mg* | 21-36% |
| P | 98-150mg* | 14-21% |
| K | 50mg | 2.50% |
| Mg | 10-41mg* | 3-14% |
| Ca | 80mg | 10% |
| Fe | 3-3.5mg* | 20-23% |
| Ze | 2.5-3.8mg* | 17-25% |

**Supplementary Table 2**. Differences in RMR and aRMR before and after the study: sensitivity analysis

|  | Parameters | | Follow up | | | | |
| --- | --- | --- | --- | --- | --- | --- | --- |
|  |  |  | Baseline | Week 4 | Week 8 | Week 12 | Week 16 |
| *Sensitivity 1* |  |  |  |  |  |  |  |
|  | Katch-McArdle^a^ determined RMR, kcal | β (95%CI) | 0 | -7.4 (-12.7, -2.1) | -14.1 (-19.5, -8.6) | -18.0 (-23.7, -12.3) | -19.4 (-25.3, -13.4) |
|  |  | adjusted *p*- value | N/A | 0.002 | <0.0001 | <0.0001 | <0.0001 |
|  | Katch-McArdle determined aRMR, kcal/kg | β (95%CI) | 0 | 0.07 (0.03, 0.11) | 0.13 (0.09, 0.18) | 0.17 (0.13, 0.21) | 0.18 (0.14, 0.23) |
|  |  | adjusted *p*- value | N/A | 0.003 | <0.0001 | <0.0001 | <0.0001 |
|  | BIA^b^ determined RMR, kcal | β (95%CI) | 0 | -13.5 (-20.5, -6.4) | -25.0, (-32.3, -17.7) | -33.7 (-41.4, -26.2) | -35.0 (-42.9, -27.2) |
|  |  | adjusted *p*- value | N/A | 0.0002 | <0.0001 | <0.0001 | <0.0001 |
|  | BIA determined aRMR, kcal/kg | β (95%CI) | 0 | -0.09 (-0.16, -0.01) | -0.14 (-0.22, -0.06) | -0.21 (-0.29, -0.13) | -0.20 (-0.29, -0.12) |
|  |  | adjusted *p*- value | N/A | 0.89 | 0.36 | 0.01 | 0.12 |
| *Sensitivity 2* |  |  |  |  |  |  |  |
| Male | Katch-McArdle^a^ determined RMR, kcal | β (95%CI) | 0 | -13.5 (-22.6, -4.4) | -17.4 (-27.2, -7.5) | -14.4 (-24.5, -4.2) | -21.1 (-32.9, -9.4) |
|  |  | adjusted *p*- value | N/A | 0.03 | 0.006 | 0.05 | 0.005 |
|  | Katch-McArdle determined aRMR, kcal/kg | β (95%CI) | 0 | 0.07 (0.03, 0.11) | 0.09 (0.04, 0.13) | 0.07 (0.03, 0.12) | 0.1 (0.04, 0.15) |
|  |  | adjusted *p*- value | N/A | 0.02 | 0.004 | 0.02 | 0.005 |
|  | BIA^b^ determined RMR, kcal | β (95%CI) | 0 | -14.9 (-26.3, -3.5) | -22.1 (-34.5, -9.8) | -21.2 (-33.9, -8.6) | -27.6 (-42.2, -13.0) |
|  |  | adjusted *p*- value | N/A | 0.08 | 0.005 | 0.01 | 0.003 |
|  | BIA determined aRMR, kcal/kg | β (95%CI) | 0 | 0.01 (-0.04, 0.06) | -0.01 (-0.06, 0.04) | -0.04 (-0.1, 0.01) | -0.02 (-0.08, 0.05) |
|  |  | adjusted *p*- value | N/A | 0.99 | 0.99 | 0.48 | 0.97 |
| Female | Katch-McArdle^a^ determined RMR, kcal | β (95%CI) | 0 | -8.0 (-13.2, -2.8) | -14.8 (-20.2, -9.4) | -19.5 (-25.0, -13.9) | -20.8 (-26.6, -15.1) |
|  |  | adjusted *p*- value | N/A | 0.03 | <0.0001 | <0.0001 | <0.0001 |
|  | Katch-McArdle determined aRMR, kcal/kg | β (95%CI) | 0 | 0.07 (0.02, 0.11) | 0.13 (0.08, 0.18) | 0.17 (0.12, 0.22) | 0.19 (0.14, 0.24) |
|  |  | adjusted *p*- value | N/A | 0.02 | <0.0001 | <0.0001 | <0.0001 |
|  | BIA^b^ determined RMR, kcal | β (95%CI) | 0 | -13.7 (-20.6, -6.8) | -25.2, (-32.4, -18.0) | -34.1 (-41.6, -26.6) | -36.0 (-43.6, -28.3) |
|  |  | adjusted *p*- value | N/A | 0.001 | <0.0001 | <0.0001 | <0.0001 |
|  | BIA determined aRMR, kcal/kg | β (95%CI) | 0 | -0.08 (-0.17, 0.01) | -0.13 (-0.22, -0.03) | -0.20 (-0.30, -0.11) | -0.19 (-0.28, -0.09) |
|  |  | adjusted *p*- value | N/A | 0.39 | 0.05 | 0.0004 | 0.002 |
| *Sensitivity 3* |  |  |  |  |  |  |  |
| 18–35 years | Katch-McArdle^a^ determined RMR, kcal | β (95%CI) | 0 | -6.5 (-13.5, 0.4) | -15.8 (-23.1, -8.6) | -18.1 (-23.1, -8.6) | -20.1 (-27.9, -12.3) |
|  |  | adjusted *p*- value | N/A | 0.03 | <0.0001 | 0.0002 | <0.0001 |
|  | Katch-McArdle determined aRMR, kcal/kg | β (95%CI) | 0 | 0.06 (0.01, 0.12) | 0.15 (0.09, 0.20) | 0.17 (0.11, 0.23) | 0.19 (0.13, 0.25) |
|  |  | adjusted *p*- value | N/A | 0.06 | <0.0001 | 0.0001 | <0.0001 |
|  | BIA^b^ determined RMR, kcal | β (95%CI) | 0 | -12.5 (-22.0, -3.1) | -27.4 (-37.3, -17.5) | -33.4 (-43.9, -22.9) | -35.0 (-45.1, -23.9) |
|  |  | adjusted *p*- value | N/A | 0.03 | <0.0001 | <0.0001 | <0.0001 |
|  | BIA determined aRMR, kcal/kg | β (95%CI) | 0 | -0.12 (-0.22, -0.02) | -0.16 (-0.27, -0.06) | -0.26 (-0.37, -0.14) | -0.18 (-0.30, -0.07) |
|  |  | adjusted *p*- value | N/A | 0.73 | 0.54 | 0.03 | 0.50 |
| 36–65 years | Katch-McArdle^a^ determined RMR, kcal | β (95%CI) | 0 | -10.8 (-19.4, -2.3) | -12.9 (-21.7, -4.1) | -22.1 (-31.2, -12.9) | -22.9 (-32.4, -13.4) |
|  |  | adjusted *p*- value | N/A | 0.01 | 0.01 | 0.0001 | 0.0003 |
|  | Katch-McArdle determined aRMR, kcal/kg | β (95%CI) | 0 | 0.09 (0.03, 0.16) | 0.11 (0.05, 0.18) | 0.19 (0.12, 0.26) | 0.20 (0.12, 0.27) |
|  |  | adjusted *p*- value | N/A | 0.09 | 0.06 | 0.002 | 0.006 |
|  | BIA^b^ determined RMR, kcal | β (95%CI) | 0 | -17.3 (-27.6, -7.0) | -22.8, (-33.6, -12.1) | -37.0 (-48.2, -25.9) | -39.5 (-50.9, -28.1) |
|  |  | adjusted *p*- value | N/A | 0.009 | 0.001 | <0.0001 | <0.0001 |
|  | BIA determined aRMR, kcal/kg | β (95%CI) | 0 | -0.04 (-0.16, 0.07) | -0.09 (-0.21, 0.04) | -0.12 (-0.25, 0.002) | -0.17 (-0.30, -0.04) |
|  |  | adjusted *p*- value | N/A | 0.99 | 0.97 | 0.86 | 0.82 |

Notes:

1. Data was presented as β (95% confidence interval).
2. Abbreviation: CI, confidence interval; RMR, resting metabolic rate; aRMR, adjusted RMR.
3. Adjusted RMR was calculated by RMR (kcal) divided by fat-free mass (kg).
4. ^a^Katch-McArdle prediction equation: RMR=370 + 21.6*lean body mass (kg).
5. ^b^BIA determined RMR based on Tanita MC-180 (Tanita Corporation, MC-180, Tokyo, Japan).
6. Changes over time were analyzed using linear mixed-effects models with time as a fixed effect, participant as a random effect, and adjusted for age, sex (where necessary), physical activity, sleep hour, dietary intake, and baseline FFM and FM, followed by post hoc comparisons between time points with Tukey-Kramer adjustment. All these parameters obtained at each follow-up visit were considered as repeated measurements.
7. Sensitivity analysis 1 was conducted in the patients excluded 2 outliers (n=129).
8. Sensitivity analysis 2 was conducted in different sexes (male ***vs.*** female=38 ***vs.*** 93).
9. Sensitivity analysis 3 was conducted in different age groups (18–35 years ***vs.***36–65 years=87 ***vs.*** 44).

**Supplementary Table 3.**  Associations between aRMR changes and age, sex, and baseline body composition: generalized linear model analysis.

| Variables | | Follow up |  |  |  |  |
| --- | --- | --- | --- | --- | --- | --- |
|  |  | Baseline | Week 4 | Week 8 | Week 12 | Week 16 |
| Diff_FFM | β, 95%CI | NA | -0.13 (-0.14, -0.12) | -0.15 (-0.16, -0.14) | -0.15 (-0.16, -0.15) | -0.14 (-0.15, -0.13) |
|  | *p*-value | NA | <0.0001 | <0.0001 | <0.0001 | <0.0001 |
| Diff_FM | β, 95%CI |  | -0.003 (-0.01, 0.006) | -0.005 (-0.01, 0.0006) | -0.005 (-0.01, -0.00003) | -0.006 (-0.01, -0.002) |
|  | *p*-value |  | 0.53 | 0.08 | 0.05 | 0.005 |
| Age | β, 95%CI |  | 0.0002 (-0.001, 0.002) | -0.0002 (-0.002, 0.002) | -0.0001 (-0.002, 0.002) | -0.0005 (-0.002, 0.001) |
|  | *p*-value |  | 0.76 | 0.84 | 0.91 | 0.58 |
| Sex, male | β, 95%CI |  | -0.01 (-0.06, 0.04) | -0.03 (-0.09, 0.02) | -0.02 (-0.08, 0.04) | -0.03 (-0.09, 0.02) |
|  | *p*-value |  | 0.64 | 0.25 | 0.49 | 0.24 |
| Baseline FFM | β, 95%CI |  | -0.0009 (-0.003, 0.002) | -0.002 (-0.004, 0.0006) | -0.002 (-0.005, 0.0005) | -0.003 (-0.006, -0.0001) |
|  | *p*-value |  | 0.44 | 0.14 | 0.1 | 0.04 |
| Baseline FM | β, 95%CI |  | 0.0003 (-0.002, 0.002) | -0.0008 (-0.003, 0.001) | -0.0004 (-0.003, 0.002) | -0.001 (-0.004, 0.001) |
|  | *p*-value |  | 0.77 | 0.46 | 0.75 | 0.27 |

Notes:

1. The difference in fat-free mass (FFM) and fat mass (FM) between different measure time and baseline (week0) was presented as diff_FFM and diff_FM.
2. Adjusted RMR (aRMR) was calculated by RMR (Katch-McArdle prediction equation) (kcal) divided by FFM (kg).
3. Katch-McArdle prediction equation: RMR=370 + 21.6*lean body mass (kg).
4. Abbreviation: FM, fat mass; FFM, free fat mass, RMR, resting metabolic rate; aRMR, adjusted RMR.


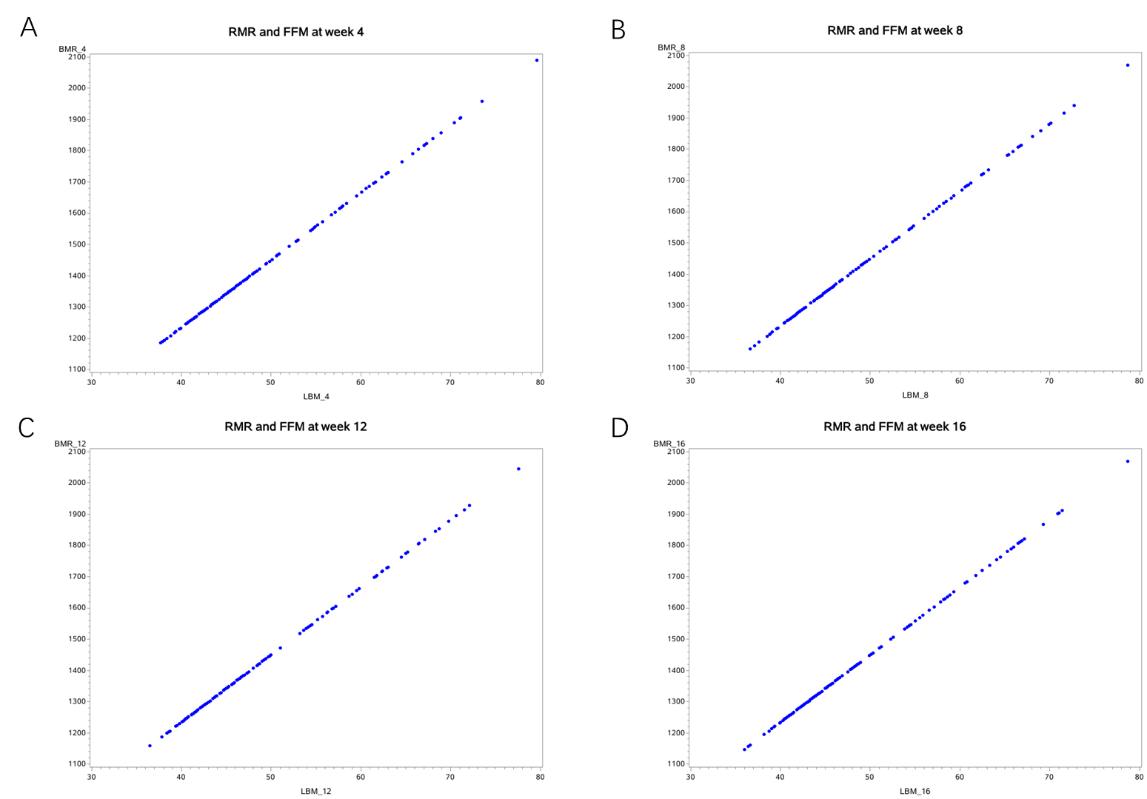


**Supplementary Figure 1:** The scatter plot of RMR and FFM: (A) the Pearson correlation between RMR and FFM at week 4; (B) the Person correlation between RMR and FFM at week 8; (C) the Person correlation between RMR and FFM at week 12; (D) the Person correlation between RMR and FFM at week 16.
